# Self-Diagnosis of Localization Status for Autonomous Mobile Robots

**DOI:** 10.3390/s18093168

**Published:** 2018-09-19

**Authors:** Jiwoong Kim, Jooyoung Park, Woojin Chung

**Affiliations:** 1 School of Mechanical Engineering, Korea University, Seoul 02841, Korea; kjw0207@gmail.com; 2 Department of Control and Instrumentation Engineering, Korea University, Sejong 30019, Korea; parkj@korea.ac.kr

**Keywords:** mobile robot, localization, light detection and ranging sensor, failure detection, support vector machine

## Abstract

It is essential to provide reliable localization results to allow mobile robots to navigate autonomously. Even though many state-of-the-art localization schemes have so far shown satisfactory performance in various environments, localization has still been difficult under specific conditions, such as extreme environmental changes. Since many robots cannot diagnose for themselves whether the localization results are reliable, there can be serious autonomous navigation problems. To solve this problem, this study proposes a self-diagnosis scheme for the localization status. In this study, two indicators are empirically defined for the self-diagnosis of localization status. Each indicator shows significant changes when there are difficulties in light detection and ranging (LiDAR) sensor-based localization. In addition, the classification model of localization status is trained through machine learning using the two indicators. A robot can diagnose the localization status itself using the proposed classification model. To verify the usefulness of the proposed method, we carried out localization experiments in real environments. The proposed classification model successfully detected situations where the localization accuracy is significantly degraded due to extreme environmental changes.

## 1. Introduction

Recently, various kinds of autonomous robots are used in the real world. Transportation robots [1] are used to improve work efficiency in environments such as factories and warehouses. Service robots [2] provide room service in a hotel. In addition, delivery robots [3] perform various tasks including food delivery service in outdoor environments. The prerequisite for achieving long-term autonomy of these robots in the real world is the development of reliable localization technology.

The purpose of localization is to estimate a global pose or local pose of a robot using sensors such as a light detection and ranging (LiDAR) sensor, a vision sensor and so forth. Autonomous mobile robots move from their current location to the target location by considering the localization results. In other words, the robot searches for an optimal path to the target location and selects an appropriate control strategy on the basis of its estimated pose. Therefore, localization is the most fundamental technology for autonomous mobile robots.

To date, many studies have evaluated localization performance based on localization errors. However, if the localization errors are maintained below a certain threshold, then they do not significantly affect the autonomous navigation in many cases. For example, if dangerous terrain is accurately detected in the local coordinate system of the robot, then safe motion control is possible even if the localization results are slightly inaccurate. In terms of practical application, it is more important to determine whether the estimated pose of the robot is reliable. If the estimated pose is not reliable, the robot may fail to perform its task or behave abnormally. Therefore, an unreliable pose should not be used to determine the navigation behavior of the robot.

State-of-the-art localization methods that use a LiDAR sensor have shown robust and reliable localization results in various environments [4,5,6,7,8]. The LiDAR sensor has the advantages of having a long measurement range and high accuracy. Thus, if the precise environmental map is given, the robot pose can be accurately estimated through the LiDAR sensor-based localization system without the help of any expensive global navigation satellite system (GNSS). Nevertheless, localization failure occasionally occurs when LiDAR sensor-based localization methods are used. Localization failure can be caused by various factors, such as extreme changes in the environment, inadequate natural landmarks and wheel slippage. However, it is difficult to prevent these factors in advance. In addition, it is also difficult to develop localization methods that are robust in all situations and environments. Therefore, it is essential to develop a technique that can diagnose the current localization status.

There have been various studies on diagnosing the localization status. Moon et al. [9] used matching errors to detect localization failure caused by an abrupt wheel slippage. If the current matching error exceeds the threshold computed from the distribution of past matching errors, then the current localization status is classified as localization failure. The method proposed by Moon et al. considered that the matching error significantly increases when wheel slippage occurs. Lee and Chung [10] defined a corrupted measurement as when the range error is more than 10%. The percentage of uncorrupted range measurements was defined as the reliability. If the reliability is below the threshold, the update phase is skipped, since the robot assumes that the estimated pose is not reliable. Self-adaptive Monte Carlo localization [11] detects localization failure on the basis of the maximum probabilities of samples. If the maximum probability of samples is below the threshold, a few of the local samples are converted to global samples. Fujii et al. [12] detected localization failures through logistic regression using the maximum probability and the standard deviation of samples. Furthermore, Fujii et al. [12] proposed a hybrid localization scheme using the probability of localization failure calculated by the logistic function.

Localization status has also been used to update the map. Sun et al. [13] diagnosed the localization status using the matching score suggested by Olson [14]. The map is updated when the matching score exceeds the threshold. In dual-timescale Normal Distribution Transform Monte Carlo Localization(NDT–MCL) [15], the map is updated when the trace of the covariance of the estimated pose is less than the threshold.

In summary, existing studies diagnose the target localization status using indicators. However, the defined indicators may be highly dependent on the localization scheme. For example, the matching error used by Moon et al. [9] and the matching score used by Sun et al. [13] are indicators obtained from each localization algorithm. Moreover, the maximum probability of samples or the covariance of the estimated pose may be less relevant to localization results, depending on the design strategy of the sensor model or the environment.

This study focuses on a localization method introduced in our previous work [16]. In Reference [16], a localization method that considers the reliability of range measurements made by a LiDAR sensor was introduced. The localization method of [16] showed satisfactory performance in environments with glass walls, dynamic obstacles and static changes. Nevertheless, there are still difficulties in environments in which extreme changes occur. Thus, the diagnosis of localization status is essential. Therefore, this study proposes a new method for the diagnosis of localization status.

In the proposed method, the localization status is classified as success or failure. The proposed method exploits the navigation experience of a robot in real environments. The navigation experience consists of two indicators that are categorized based on the true localization status. The first indicator exploits the reliability of the range measurement introduced in Reference [16]. We focus on distance errors of range measurements, for which the reliability is relatively high. The second indicator exploits the estimated heading of the robot. The feasibility of the estimated heading is verified by considering the motion uncertainty and the angular displacement of the odometry. In our method, the classification model for the localization status is trained through machine learning using two indicators. In the next section, we give detailed descriptions of each indicator.

The rest of this paper is organized as follows: In Section 2, we present the localization status indicators, which are empirically defined. Section 3 illustrates the proposed classification model for the localization status. The training and test results of the proposed classification model are presented in Section 4. The concluding remarks will be presented in Section 5.

## 2. Localization Status Indicators

### 2.1. Distance Errors of Range Measurements

In our previous work [16], a localization method using a LiDAR sensor was introduced. In Reference [16], the reliability of every range measurement is considered to reduce the effects of distance errors caused by glass walls, dynamic obstacles and static changes in environments. The reliability of each range measurement is computed by considering the distance error computed based on the known map information. Thus, if the distance error of any range measurement is relatively large because of unknown obstacles or unexpected reflections, the reliability of that range measurement becomes low. After computing the reliability for every range measurement, only relatively reliable range measurements are used for localization. Therefore, the localization method of [16] is robust to partial distance errors and the robot pose can be successfully estimated using some reliable range measurements. The first indicator is defined based on the reliability of the range measurement introduced in Reference [16].

**Figure 1:**
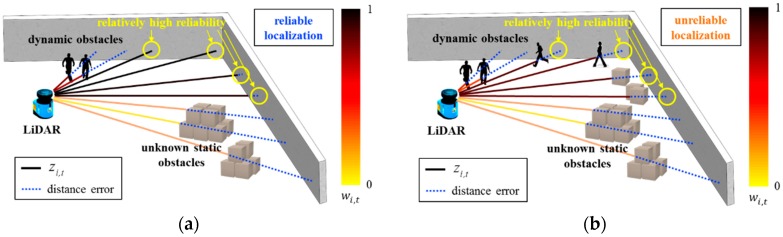
Reliability and distance error of range measurements. (**a**) Reliable localization; (**b**) Unreliable localization. The length of the solid lines represents $z_{i,t}.$ The color of the solid lines represents $w_{i,t}.$ The length of the blue dotted line represents the distance error of each range measurement.

In Figure 1, range measurements denoted with yellow circles exhibit relatively high reliability. The length of the blue dotted line represents the distance error of each range measurement. In Figure 1a, although the distance error calculated for all range measurements is large, the distance error calculated for range measurements denoted by yellow circles is small. In other words, range measurements in which the reliability is relatively high are accurate. Thus, localization can be performed successfully. Conversely, as shown in Figure 1b, although the range measurements denoted with yellow circles exhibit relatively high reliability, their distance errors are large. Consequently, the localization result is unreliable.

Therefore, this study assumes that localization can be performed successfully if a small mean distance error is calculated for range measurements with a relatively high reliability. Let *D_t_* be the mean distance error of range measurements with a reliability corresponding to the upper τ%. *D_t_* is defined as follows:

(1) $$D_{t}=\frac{\sum_{i\in I}\left|z_{i,t}-z_{i,t}^{*}\right|}{n^{\prime }},$$

$z_{i,t}$ is the distance measured by the *i*th scan of the LiDAR sensor at time *t*, $z_{i,t}^{*}$ is the *i*th reference distance calculated from the map at time *t*, $n^{\prime }$ is an integer corresponding to τ% of the number of total range measurements and *I* is a set of *i* of range measurements with a reliability corresponding to the upper τ%. An increase in τ increases the number of range measurements used to calculate *D_t_*. As a result, *D_t_* may increase even if the localization error does not actually increase. Conversely, a decrease in τ results in insensitivity to a situation in which the localization error actually increases.

In addition, it is important to note that the localization error increases cumulatively. Thus, when *D_t_* is large, the localization error may increase continuously. Furthermore, although *D_t_* suddenly approaches 0, the localization error does not immediately approach 0. In conclusion, the localization error is also affected by the past value of *D_t_*. Therefore, we define *ED_t_* as follows:

(2) $$ED_{t}=\{\begin{array}{ll} D_{1}, & if\;\left(t=1\right),\\ \lambda D_{t}+\left(1-\lambda \right)ED_{t-1}, & if\;\left(t>1\right). \end{array}$$

In Equation (2), *ED_t_* is computed using an exponentially weighted moving average of *D_t_* and λ is a weight for the current value of *D_t_*. Consequently, *ED_t_* considers past values of *D_t_*. We use *ED_t_* as the first indicator for diagnosing the localization status.

**Figure 2:**
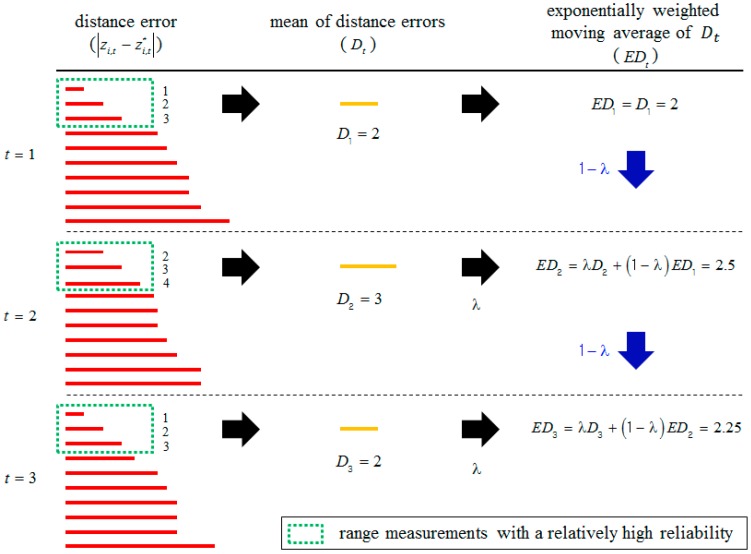
Examples of *ED_t_* computation. The length of the red solid lines represents the distance error of each range measurement. In these examples, it is assumed that λ is 0.5.

Figure 2 shows examples of *ED_t_* computation. In Figure 2, it is assumed that the number of total range measurements is 10 at each time step and the length of the red solid lines represents the distance error of each range measurement. The range measurements with a relatively high reliability are denoted by green dotted rectangles. At time *t* = 1, the mean distance error *D*_1_ of range measurements with a relatively high reliability is computed as 2 by Equation (1). Since *ED_t_* is equal to *D_t_* at time *t* = 1, *ED*_1_ becomes 2. At time *t* = 2, *D_t_* is 3 because the distance errors of range measurements with a relatively high reliability are 2, 3 and 4, respectively. *ED_t_* is affected by both the past value and the current value of *D_t_* at time *t* = 2. As a result, *ED*_2_ becomes 2.5 by Equation (2).

### 2.2. Estimated Heading

**Figure 3:**
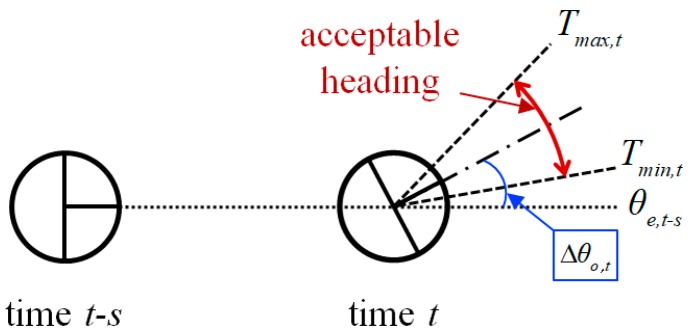
Illustration of the acceptable heading.

The second indicator exploits the estimated heading of the robot. In Figure 3, the boundary of an acceptable heading is computed by considering the motion uncertainty of the robot and the angular displacement of the odometry. It is highly likely that an abnormal localization has occurred if the estimated heading deviates from the acceptable boundary of the heading. We define *A_t_* as follows:

(3) $$A_{t}=\{\begin{array}{ll} \theta_{e,t}-T_{max,t}, & \;if\;\left(\theta_{e,t}>T_{max,t}\right),\;\\ \;0, & \;if\;\left(T_{min,t}\leq \theta_{e,t}\leq T_{max,t}\right),\\ T_{min,t}-\theta_{e,t}, & \;if\;\left(\theta_{e,t}<T_{min,t}\right), \end{array}$$

(4) $$T_{max,t}=\theta_{e,t-s}+\Delta \theta_{o,t}+\alpha \left|\Delta \theta_{o,t}\right|,$$

(5) $$T_{min,t}=\theta_{e,t-s}+\Delta \theta_{o,t}-\alpha \left|\Delta \theta_{o,t}\right|,$$

(6) $$\Delta \theta_{o,t}=\theta_{o,t}-\theta_{o,t-s}.$$

$\theta_{e,t}$ is the estimated heading at time *t*, $\theta_{o,t}$ is the odometry heading at time *t*, *T_max,t_* and *T_min,t_* are the maximum and the minimum acceptable heading at time *t*, respectively, and α is a parameter that considers the motion uncertainty of the robot.

In Equation (3), if estimated heading $\theta_{e,t}$ is within the acceptable boundary of the heading, then *A_t_* becomes 0. Otherwise, *A_t_* represents the extent to which $\theta_{e,t}$ deviates from the acceptable boundary of the heading. When the acceptable boundary of the heading is computed in Equations (4)–(6), we consider changes in the heading over a certain period of time to reduce the effect of an instantaneous angular error.

In a manner similar to *D_t_*, the localization error is also affected by the past value of *A_t_*. Therefore, *EA_t_* is defined as follows:

(7) $$EA_{t}=\{\begin{array}{ll} A_{1}, & \;if\;\left(t=1\right),\\ \lambda A_{t}+\left(1-\lambda \right)EA_{t-1}, & \;if\;\left(t>1\right). \end{array}$$

We use *EA_t_* as the second indicator for diagnosing the localization status.

**Figure 4:**
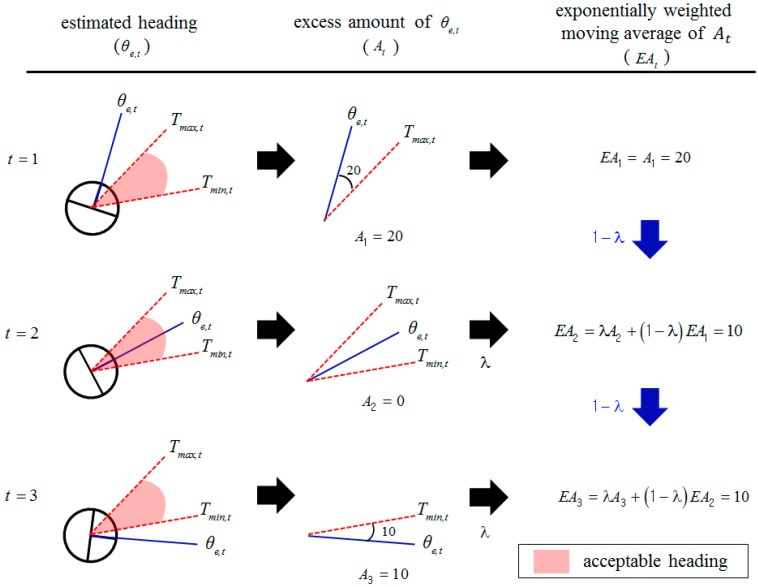
Examples of *EA_t_* computation. The blue solid lines represent estimated heading $\theta_{e,t}.$ The red dotted lines represent acceptable heading boundary *T_max,t_* and *T_min,t_*. In these examples, it is assumed that λ is 0.5.

Examples of *EA_t_* computation are shown in Figure 4. In Figure 4, since estimated heading $\theta_{e,t}$ exceeds *T_max,t_* by 20 degrees at time *t* = 1, *A*_1_ becomes 20 by Equation (3). On the other hand, *A*_2_ is 0 at time *t* = 2 because estimated heading $\theta_{e,t}$ is within the acceptable heading boundary as shown in Figure 4. At time *t* = 2, since *EA_t_* is affected by both the past value and the current value of *A_t_*_,_ Consequently, *EA*_2_ becomes 10 by Equation (7).

## 3. Diagnosis of Localization Status

### 3.1. Supervised Learning Algorithm

Supervised learning [17] is a machine learning algorithm that uses labeled training data. Thus, the training data in supervised learning consists of pairs with an input value and a desired output value. The purpose of supervised learning is to find a function that maps input values to output values. Supervised learning is divided into regression problems and classification problems, depending on the type of output data. Supervised learning is a regression problem when the output variables are continuous. In contrast, supervised learning is a classification problem when the output variables are categorical. The purpose of this study is to classify the localization status using localization status indicators. Hence, the classification model for the localization status can be trained through supervised learning if there are indicators that are categorized according to the true localization status.

### 3.2. Categorization of Localization Status Indicators

To categorize *ED* and *EA* according to the true localization status, classification rules for the true localization status are required. In the proposed method, the classification rules for the true localization status are as follows:

- If the current position error or heading error exceeds a predetermined threshold, the current localization status is classified as failure.
- If the current localization status does not correspond to failure, then the current localization status is classified as success.

If the localization error is maintained below a certain threshold, then the localization error does not have a significant effect on the autonomous navigation in many cases. Thus, the localization status is classified according to whether the localization error exceeds a predetermined threshold. The threshold of the localization error depends on the task and the environment.

In the proposed method, the localization status is classified as success or failure. Therefore, the values of *ED* and *EA* categorized as success or failure can be used as training data. In conclusion, this study defines the diagnosis of the localization status as a binary classification problem using supervised learning.

### 3.3. Classification of Localization Status using a Support Vector Machine

Various algorithms including *k*-nearest neighbors [18], logistic regression [19] and a support vector machine (SVM) [20] can be used for binary classification problems using supervised learning. We consider the following three conditions in selecting a classification algorithm.

- The classification time should be short for real-time applications.
- Overfitting should be avoided.
- It should be capable of dealing with input data that is not linearly separable.

The classification of localization status should be performed in real-time while the robot is moving. Thus, it is important to ensure the classification time is short, because other algorithms, such as localization and obstacle detection, also contribute to the overall computation time. Additionally, the classification model should avoid overfitting the training data. An over fitted classification model may exhibit poor performance for new data. Furthermore, the classification algorithm should be able to classify input data that cannot be linearly separated because our input data are not linearly separable.

Support vector machine [20] is a representative machine learning algorithm for data classification. Although SVM may require a long time to learn, depending on the input data, it can quickly classify new data by using the learned model. Moreover, overfitting can be avoided in SVM by adjusting the parameters. Furthermore, SVM is advantageous for input data that cannot be linearly separated if kernel tricks are used properly. Therefore, we exploit SVM to train the classification model of localization status.

**Figure 5:**
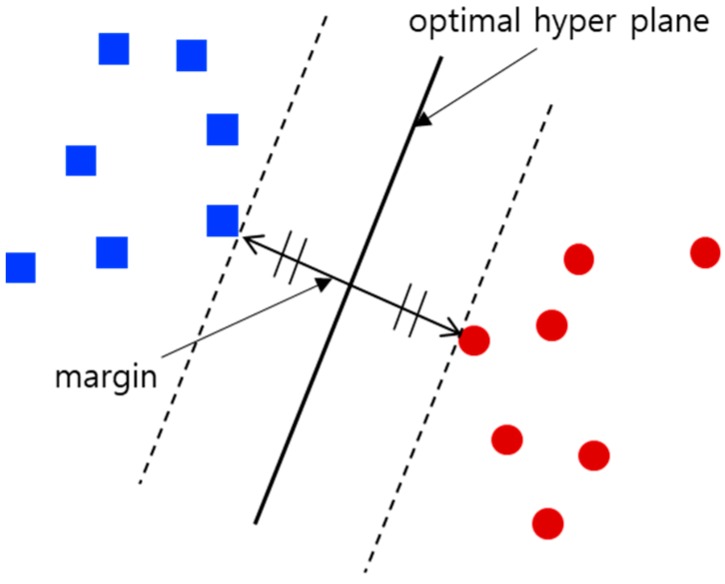
Illustration of the optimized hyperplane and the margin.

**Figure 6:**
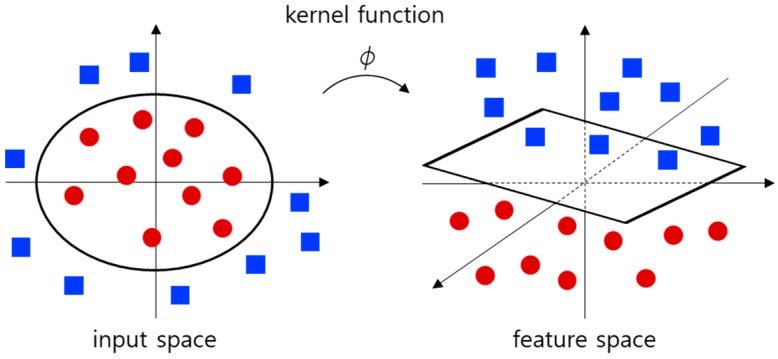
Kernel trick for mapping from an input space to a feature space.

The purpose of SVM is to find an optimized hyperplane that maximizes the margin, as shown in Figure 5. The margin indicates the distance between the optimized hyperplane and the nearest training data. Since it is difficult to classify our input data linearly in an input space, the kernel trick is used. Figure 6 shows an example in which the input space is mapped to the feature space using the kernel trick. It is possible to compute an optimized hyperplane in a high-dimensional feature space using the kernel trick. The proposed method uses a radial basis function kernel, which is suitable for many types of data because it can map data to an infinite-dimensional space.

### 3.4. Self-Diagnosis System of Localization Status

**Figure 7:**
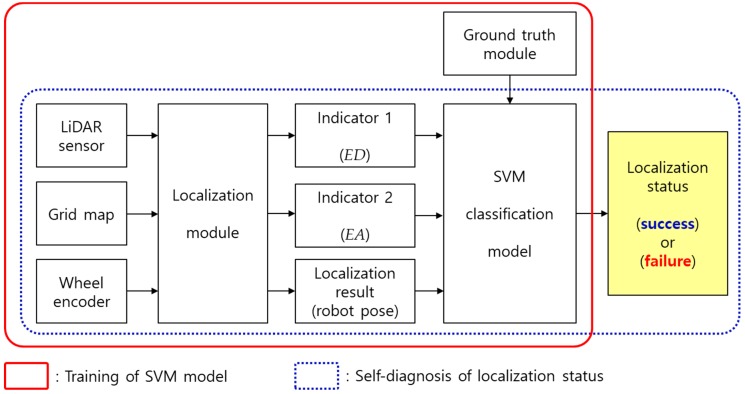
Self-diagnosis system of the localization status. SVM: support vector machine.

For long-term autonomy of robots, fault detection is an important issue [21,22]. In this section, our localization self-diagnosis system is introduced in detail. Figure 7 shows the schematic diagram of our system. There are two main procedures in this system for the diagnosis of the localization status. The first procedure is to train the SVM model and the other is to classify the localization status of the robot using the trained SVM model.

In Figure 7, the part denoted by the red solid rectangle corresponds to the training of the SVM model. In this study, the SVM model is trained based on the navigation experience of a robot. Specifically, the sensor data is collected while the robot equipped with the LiDAR sensor and the wheel encoder is driving in the environment of which grid map is given. Then, based on the sensor data and the map, the values of *ED* and *EA* that are two indicators defined in the previous section are computed in the localization module. In this procedure, it is important to know the true localization status of the moment when the *ED* and *EA* values are collected.

In this study, the ground truth module is used to find the true localization status. The ground truth module can accurately compute the true robot pose by detecting the artificial landmarks placed in the environment. Artificial landmarks used in the proposed method are reflectors with higher reflectivity than the surrounding objects. The true localization status of the robot can be found by comparing the robot pose estimated by the localization module and the true robot pose provided by the ground truth module. Consequently, the values of two indicators categorized according to the true localization status are collected. After collecting this data, the SVM classification model is trained offline.

The self-diagnosis procedure of the localization status using the trained SVM classification model is marked with a blue dotted rectangle in Figure 7. The robot computes the values of the two indicators using sensor data obtained at each moment and map information. Then, by using only the values of the two indicators as inputs to the SVM classification model, the localization status of the robot can be diagnosed. An important difference from the SVM model learning procedure is that the ground truth module is not needed. In other words, the robot can diagnosis the localization status by itself using only the data obtained through the on-board sensor, without any environmental maintenance such as the placement of artificial landmarks.

## 4. Experiments

### 4.1. Experimental Setup

**Figure 8:**
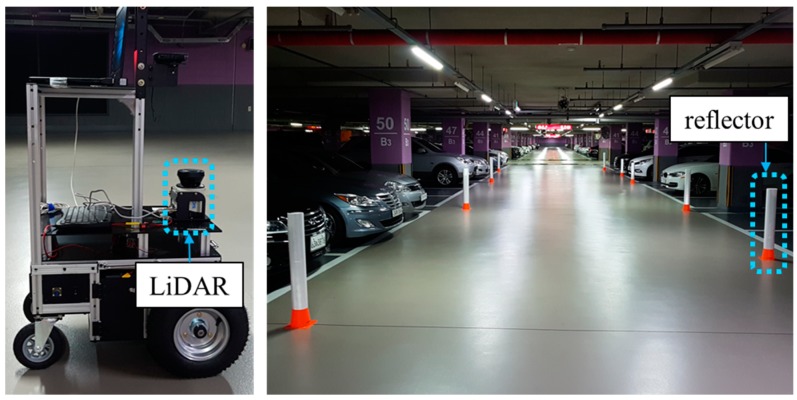
Mobile robot platform and the parking lot where the localization experiments were performed. To calculate the ground truth, the reflectors were placed in the environment.

In order to train and test the classification model for the localization status, localization experiments were performed in real environments. A two-wheeled differential-drive mobile robot equipped with a LMS100 LiDAR manufactured by SICK in Germany was used, as shown in Figure 8. The SICK LMS100 is a 2D LiDAR sensor that provides a 270° field of view with a maximum range of 20 m. Since most of the commercially available LiDAR sensors show measurement accuracy of less than 3 cm [23,24,25], there is little difference in measurement accuracy. Furthermore, most LiDAR sensors can be used in the outdoor environments because they are robust to sunlight and illumination conditions [23,24,25,26]. Nevertheless, some low-cost LiDAR sensors have too low accuracy and are not available in bright outdoor environments. However, this study assumes that the use of LiDAR sensors with too low performance is not considered. In addition, in extreme situations where it is difficult to use the LiDAR sensor due to weather conditions such as fog and heavy rain, other sensors should be used. Therefore, this study also does not consider such extreme weather conditions.

In the experiments, it is important that there are changes in the localization status. Thus, experiments were performed in a parking lot, as shown in Figure 8. In the parking lot, there are many changes in which cars are parked there over time. As a result, the localization performance can be significantly degraded. We collected sensor data over time and used them for offline mapping.

**Figure 9:**
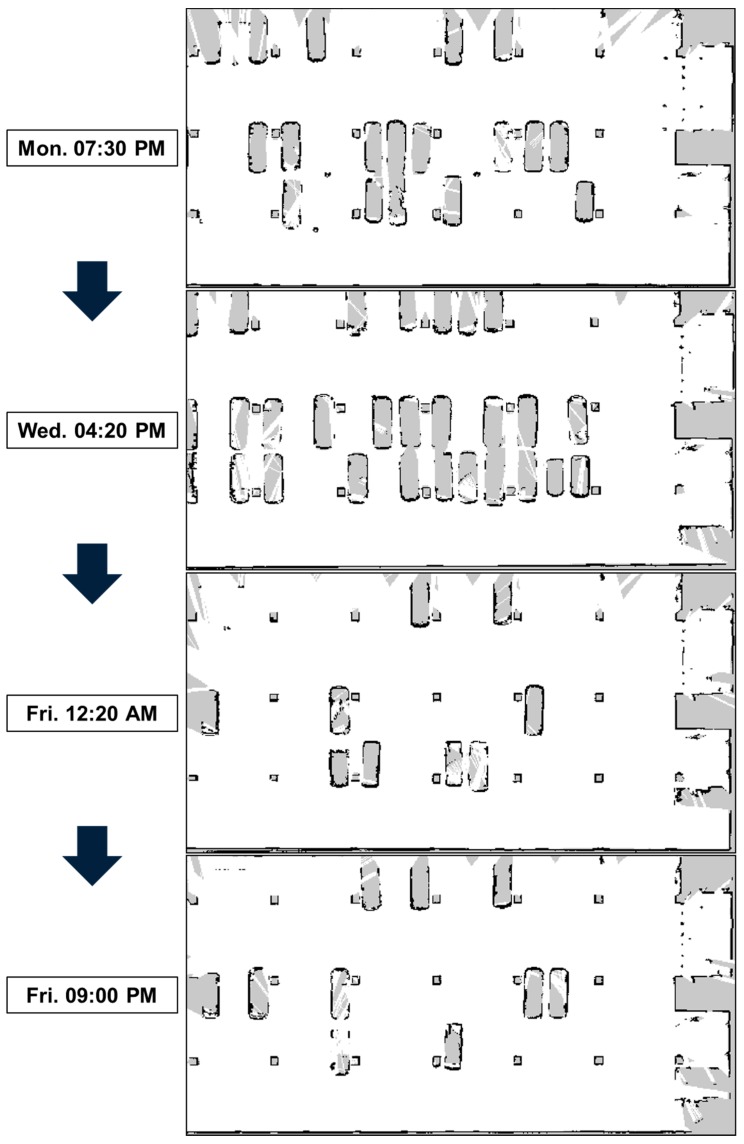
Grid maps of the parking lot showing the changes over time.

Figure 9 shows that the grid maps of the parking lot change over time. There are significant differences among the maps, owing to changes in which cars are parked there. To consider environmental changes, the data we used for the localization was different from the data we used for mapping. For example, we performed localization experiments using data collected on Wednesday afternoon with a map that was built from data collected on Friday evening.

### 4.2. Relationship between Localization Errors and the Indicators

**Figure 10:**
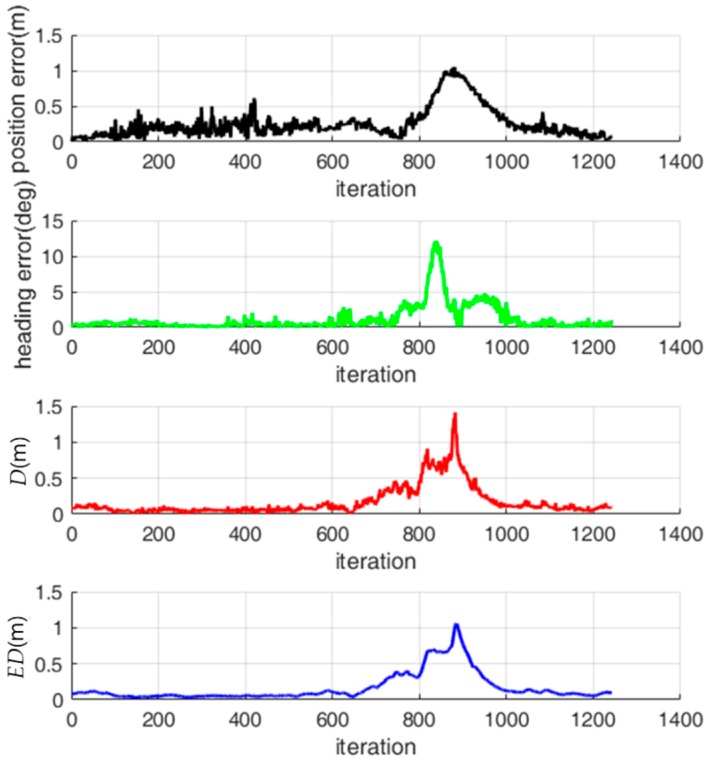
History of the localization error, *D* and *ED*.

The localization errors and localization status indicators were calculated through offline localization simulations using collected sensor data. Figure 10 shows the history of the localization errors, *D* and *ED*, while the robot is moving. *ED* is relatively large from about the 800th iteration to the 1000th iteration and the localization error is also relatively large in those iterations. Therefore, the value of *ED* is related to the localization error.

**Figure 11:**
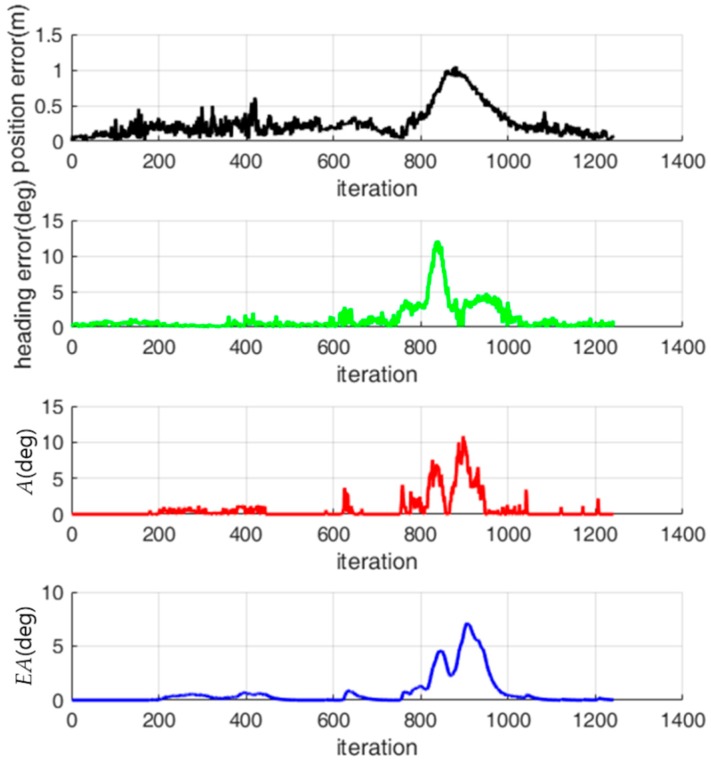
History of the localization error, *A* and *EA*.

Figure 11 shows the history of the localization error, *A* and *EA* while the robot is moving. In Figure 11, the localization error is large near the 860th iteration. However, *A* is close to 0 near the 860th iteration. On the other hand, *EA* is relatively large near the 860th iteration because *EA* is affected by past values of *A*. This indicates that *EA* is a better indicator for predicting the localization error than *A*.

### 4.3. Training Results

To categorize the localization status indicators, we set the thresholds for the position error and the heading error as 0.5 m and 5°, respectively. The localization status indicators are categorized as failure when the position error or the heading error exceeds the relevant threshold.

The classification model for localization status was trained using a data set in which *ED* and *EA* are categorized according to the true localization status. *k*-fold cross validation was used to help prevent overfitting of the classification.

**Figure 12:**
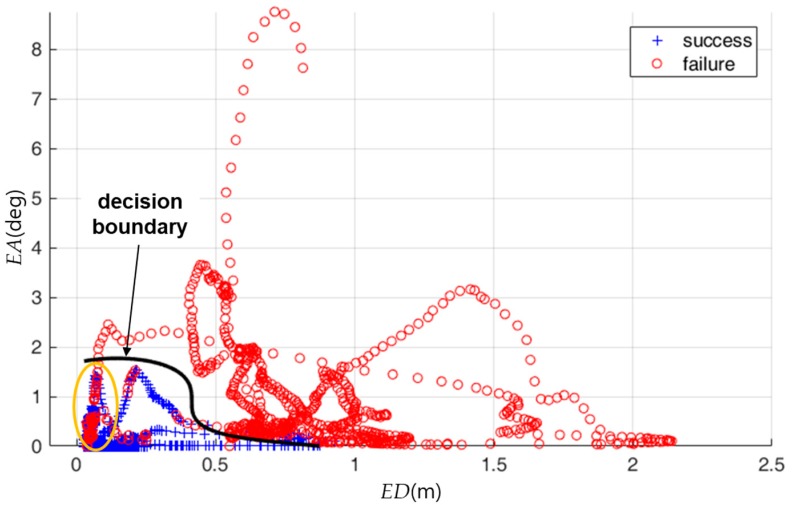
Distribution of the true localization status for the training data and the decision boundary computed for the training data. The *x*-axis is *ED*. The *y*-axis is *EA*. The black curve is the decision boundary.

Figure 12 shows the distribution of the true localization status and the decision boundary that is computed for the training data. In Figure 12, the localization status was classified as failure when either *ED* or *EA* were relatively high. The localization status was correctly classified in many cases although it was partially misclassified as shown by the yellow ellipse in Figure 12.

Table 1 shows the summarized classification results. For the training data, 94% of the data wherein true status is success were classified as success and 89% of the data wherein true status is failure were classified as failure. These results show that the proposed classification model effectively classifies the localization status for the training data.

**Figure 13:**
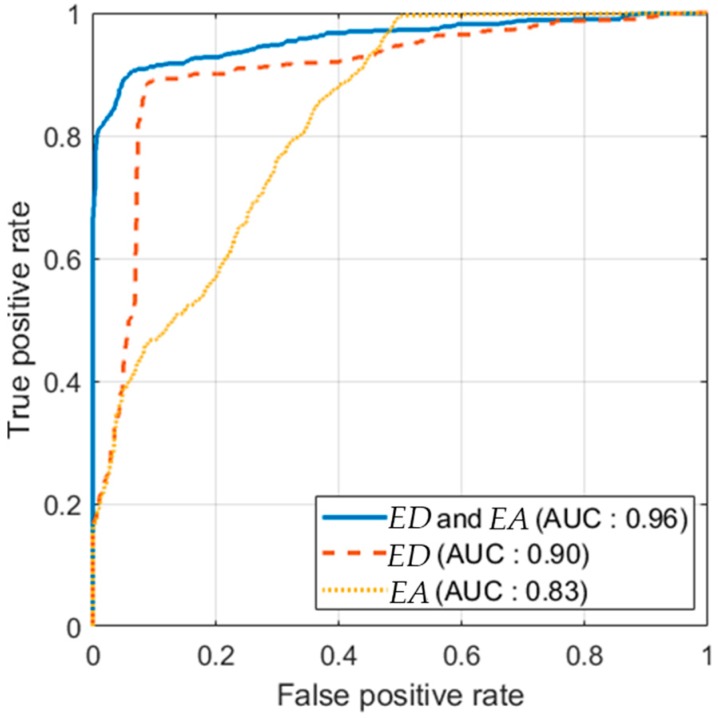
Receiver operating characteristic (ROC) curve for the proposed classifier. AUC: area under the ROC curve.

In order to verify the classification performance for each localization status indicator, we plotted the receiver operating characteristic (ROC) curve, as shown in Figure 13. In the ROC curve, when the curve is near the upper left corner, the classification model has a high true positive rate and a low false positive rate. Thus, the resultant classification model is accurate when the curve is near the upper left corner. The closeness between the curve and the upper left corner is quantitatively evaluated by the area under the ROC curve (AUC). The AUC was 0.90 for *ED*, 0.83 for *EA* and 0.96 for *ED* and *EA*. In conclusion, using both *ED* and *EA* exhibited the most accurate classification performance.

The ROC curve can also be used to determine the decision boundary. In the proposed method, the classification of success as well as the classification of failure is also important. False alarms for localization failure can considerably reduce the localization efficiency. Therefore, in the proposed method, the decision boundary was determined by maximizing the sum of the true positive rate and the true negative rate in the ROC curve.

### 4.4. Classification with Test Data

**Figure 14:**
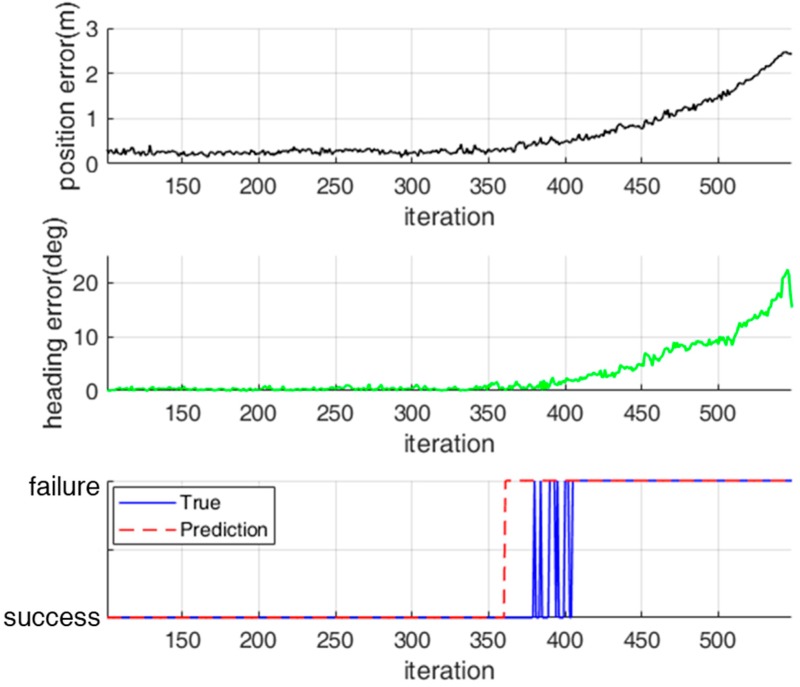
Classification of localization status for test data set 1.

**Figure 15:**
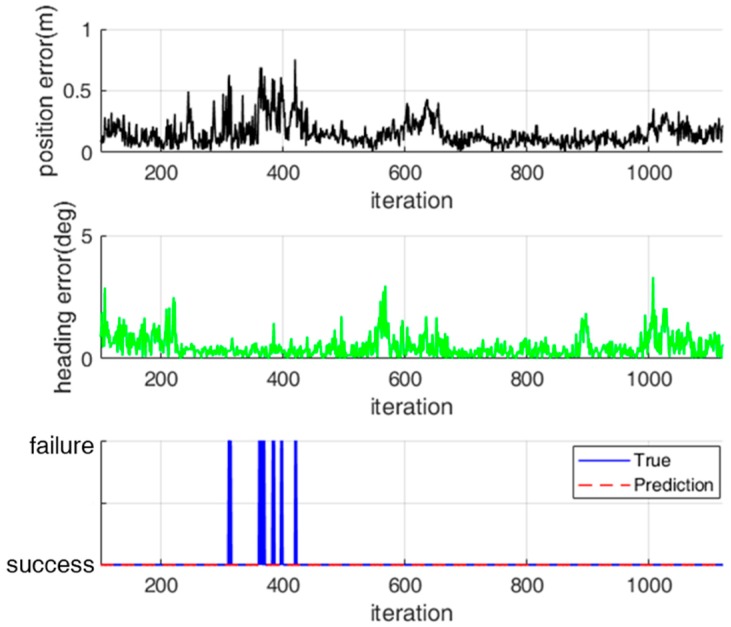
Classification of localization status for test data set 2.

For a practical application of the classification model, we evaluated the performance of the classification model for test data that were not used for training. Figure 14 and Figure 15 show the classification results for the localization status over time for two test data sets. For test data set 1, localization failure occurs continuously. In contrast, for test data set 2, localization failure occurs temporarily because the localization error increases temporarily.

For test data set 1, the localization status was successfully classified in most cases, as shown in Figure 14. However, there were some false alarms in which a true success was classified as a failure. These false alarms occurred because the observation likelihood was fortunately high near the true pose of the robot although most range measurements were erroneous. For test data set 2—as shown in Figure 15—there were some missed detections in which a true failure was classified as a success. Nevertheless, these missed detections have no significant influence on the navigation performance because the localization errors were temporarily increased.

**Figure 16:**
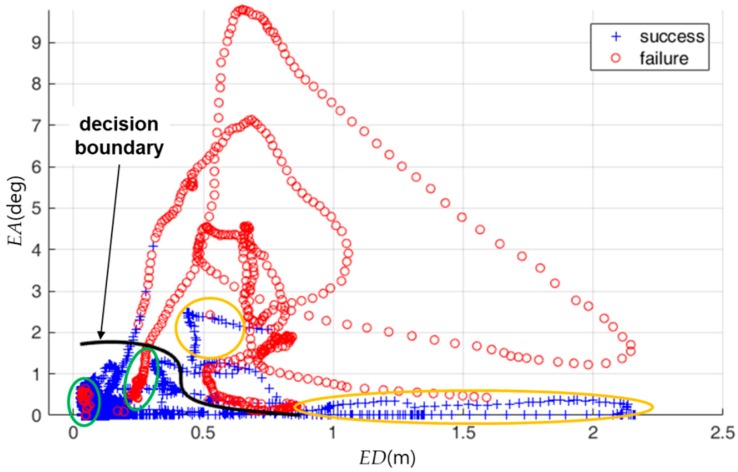
Distribution of the true localization status for the entire test data set and the decision boundary computed for the training data. The *x*-axis is *ED*. The *y*-axis is *EA*. The black curve is the decision boundary.

Figure 16 and Table 2 show the classification results of the localization status for the entire test data set. In Figure 16, yellow ellipses show the examples of false alarms, in which a true success was classified as a failure. The green ellipses show the examples of missed detections, in which a true failure was classified as a success. The proposed classification model successfully classified 86% of the total failures and 92% of the total successes. About 14% of the data in the entire data set corresponds to a localization failure. Therefore, about 2% of the data in the entire data set corresponds to a missed detection of a localization failure.

It is also important to consider the computation time required to classify the localization status. In the proposed method, we need the reliability of each range measurement to compute *ED*. However, since the reliability of each range measurement is computed in the localization algorithm, there is little additional computational cost. In addition, the computational cost of the learned SVM classification model is low because the input data and the kernel function are simple. Therefore, the real-time classification of localization status is possible.

## 5. Conclusions

To date, autonomous navigation technology of mobile robots has been developed rapidly. An important issue remaining in the commercial use of mobile robots in the real world is to guarantee the reliability of autonomous navigation technology. The long-term autonomy of the autonomous mobile robot can be realized only if the reliability of the autonomous navigation technology is ensured. Consequently, the roles of people in many fields can be replaced by autonomous mobile robots.

In order to achieve reliable autonomous navigation of mobile robots, this study proposed a self-diagnosis method for the localization status by exploiting the navigation experience of a robot in real environments. In the proposed model, the localization status was classified as a success or a failure based on two indicators. Support vector Machine, which is a supervised learning algorithm, was exploited to train the classification model for the localization status. The classification model showed satisfactory classification performance for the training data and the test data collected in the real environments. In conclusion, we verified that our indicators, which were empirically defined, can be used in the self-diagnosis of the localization status in real environments.

In this study, the proposed classification model was used to diagnose the localization status of mobile robots. However, the proposed classification model can also be applied to the localization of other platforms such as autonomous vehicles equipped with a LiDAR sensor. Therefore, it is expected that the proposed method can be used to improve the localization performance in various fields.

## Figures and Tables

**Table 1:** Confusion matrix for training data set.

	Success (Prediction)	Failure (Prediction)
Success (true)	992 (94%)	60 (6%)
Failure (true)	83 (11%)	680 (89%)

**Table 2:** Confusion matrix for test data set.

	Success (Prediction)	Failure (Prediction)
Success (true)	2958 (92%)	257 (8%)
Failure (true)	76 (14%)	461 (86%)
